# Neurofibromatosis type 2 predisposes to ependymomas of various localization, histology, and molecular subtype

**DOI:** 10.1007/s00401-021-02304-4

**Published:** 2021-04-12

**Authors:** Catena Kresbach, Mario M. Dorostkar, Abigail K. Suwala, Annika K. Wefers, Leonille Schweizer, Lara Engertsberger, Brigitte Bison, Martin Mynarek, Katja Kloth-Stachnau, Michael Spohn, Andreas von Deimling, Martin Benesch, Christian Hagel, Viktor-F. Mautner, Stefan Rutkowski, Ulrich Schüller

**Affiliations:** 1 Department of Pediatric Hematology and Oncology, University Medical Center Hamburg-Eppendorf, Hamburg, Germany; 2 Research Institute Children’s Cancer Center Hamburg, Martinistrasse 52, N63 (HPI), 20251 Hamburg, Germany; 3 Center for Neuropathology, Ludwig-Maximilians-University, Munich, Germany; 4 German Center for Neurodegenerative Diseases, Munich, Germany; 5 Department of Neuropathology, Institute of Pathology, University of Heidelberg, Heidelberg, Germany; 6 Clinical Cooperation Unit Neuropathology, German Consortium for Translational Cancer Research (DKTK), German Cancer Research Center (DKFZ), Heidelberg, Germany; 7 Institute of Neuropathology, University Medical Center Hamburg-Eppendorf, Hamburg, Germany; 8 Institute for Neuropathology, Charité Universitätsmedizin Berlin, Berlin, Germany; 9 Division of Pediatric Hematology and Oncology, Department of Pediatrics and Adolescent Medicine, Medical University of Graz, Graz, Austria; 10 Department of Diagnostic and Interventional Neuroradiology, University Hospital Augsburg, Augsburg, Germany; 11 Bioinformatics Core, University Medical Center Hamburg-Eppendorf, Hamburg, Germany; 12 Department of Neurology, University Medical Center Hamburg-Eppendorf, Hamburg, Germany

Neurofibromatosis Type 2 (NF2) is a tumor predisposition syndrome resulting from inactivating alterations in the *NF2* gene. Patients typically develop multiple neoplastic and dysplastic lesions, predominantly in the nervous system. Apart from schwannoma and meningioma, ependymoma belongs to the typical tumor spectrum of these patients. Sporadic ependymomas encompass multiple clinically relevant subgroups based on localization, genetic alterations as well as epigenetic and transcriptomic tumor profiles [3]. However, the spectrum of ependymomas in patients with NF2 is less clear. Open questions are, whether NF2-associated ependymomas are strictly limited to the spinal cord, which molecular subgroups they encompass, and how they may be distinguished from sporadic cases. Here, we present data from 33 NF2-associated ependymomas (Table 1). In-line with previous studies [2, 4], most tumors were located in the spinal cord, but often lacked typical pseudorosettes (Suppl. Fig. 1, online resource). However, we also identified 6 intracranial cases (cases 1–6), 3 of them arising distant from the medulla oblongata as suggested by MRI (cases 1, 2, and 6, Fig. 1, Suppl. Fig. 2, online resource). NF2 patients with intracranial tumors were 10.9 years old on average, as compared to 19.4 years in NF2 patients with spinal ependymoma (SP-EPN) and 41 years in patients with SP-EPN without reported NF2 [3]. In part, intracranial tumors displayed signs of anaplasia and loss of H3K27 trimethylation (Fig. 1, Suppl. Fig. 3, online resource), had to be treated aggressively, and resulted in the patient’s death (Suppl. Table 1, online resource). DNA methylation profiling and application of the brain tumor classifier [1] identified a significant match for posterior fossa ependymoma, group B (PF-EPN-B) for case 3. Case 5 that was attached to the medulla oblongata matched the methylation class of spinal ependymoma (SP-EPN). Three other intracranial cases remained without significant match (Table 1, for *t*-SNE analysis, see Fig. 1m). All 14 SP-EPN with available molecular data clearly fell into the methylation class of SP-EPN. However, copy number variation profiles of NF2-associated SP-EPN showed a rather flat genome compared to sporadic SP-EPN (Fig. 1n). Together, our data indicate that the spectrum of CNS tumors in NF2 patients includes ependymomas of different types and localizations.

**Table 1:**
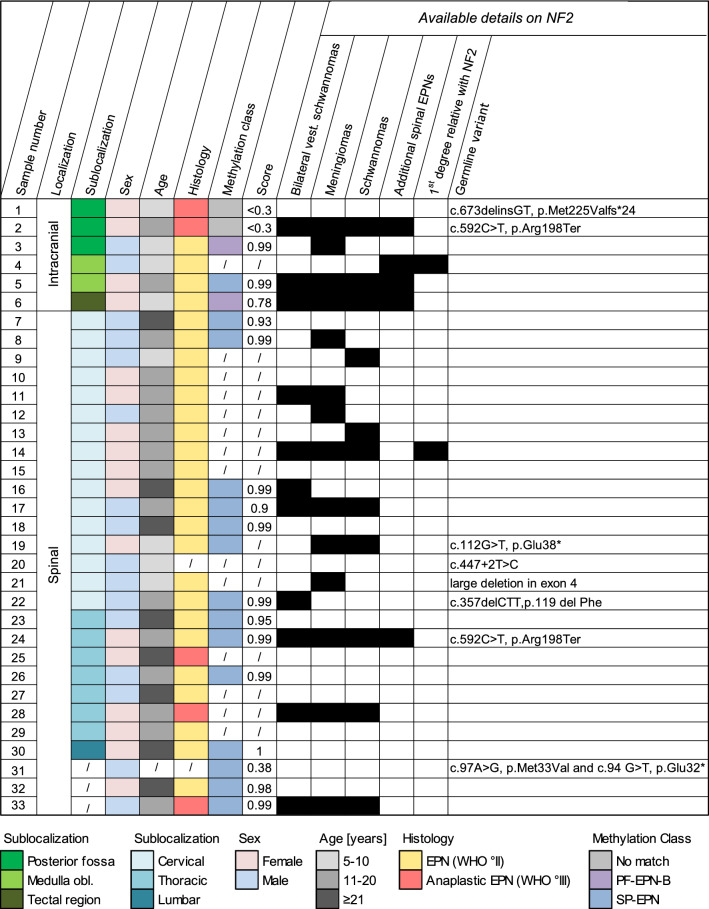
Data overview table over 33 NF2-associated ependymomas

All patients were diagnosed with NF2. One patient was diagnosed with a SP-EPN at the age of 11 and an intracranial EPN in the fossa posterior at the age of 15 (samples 24 and 2, respectively). Additional patient data relevant to the NF2 diagnosis were collected whenever available: reported occurrence of other NF2-associated tumors is depicted as a black square. For 8 cases, genetic profiling was accessible. Germline variants were detected after alignment with the RefSeq transcript NM_000268.3 (cases 19, 22, 31), NM_000268.4 (cases 2, 20, 24) or NM_181831.2 (case 1). Score = calibrated score from version 11bv4 of the DKFZ brain tumor classifier (www.molecularneuropathology.org)

*EPN* ependymoma, *i. ventr.* intraventricular, *NF2* neurofibromatosis type 2, *PF-EPN-B* posterior fossa ependymoma group B, *SP-EPN* spinal ependymoma, *vest.* vestibular

**Fig. 1:**
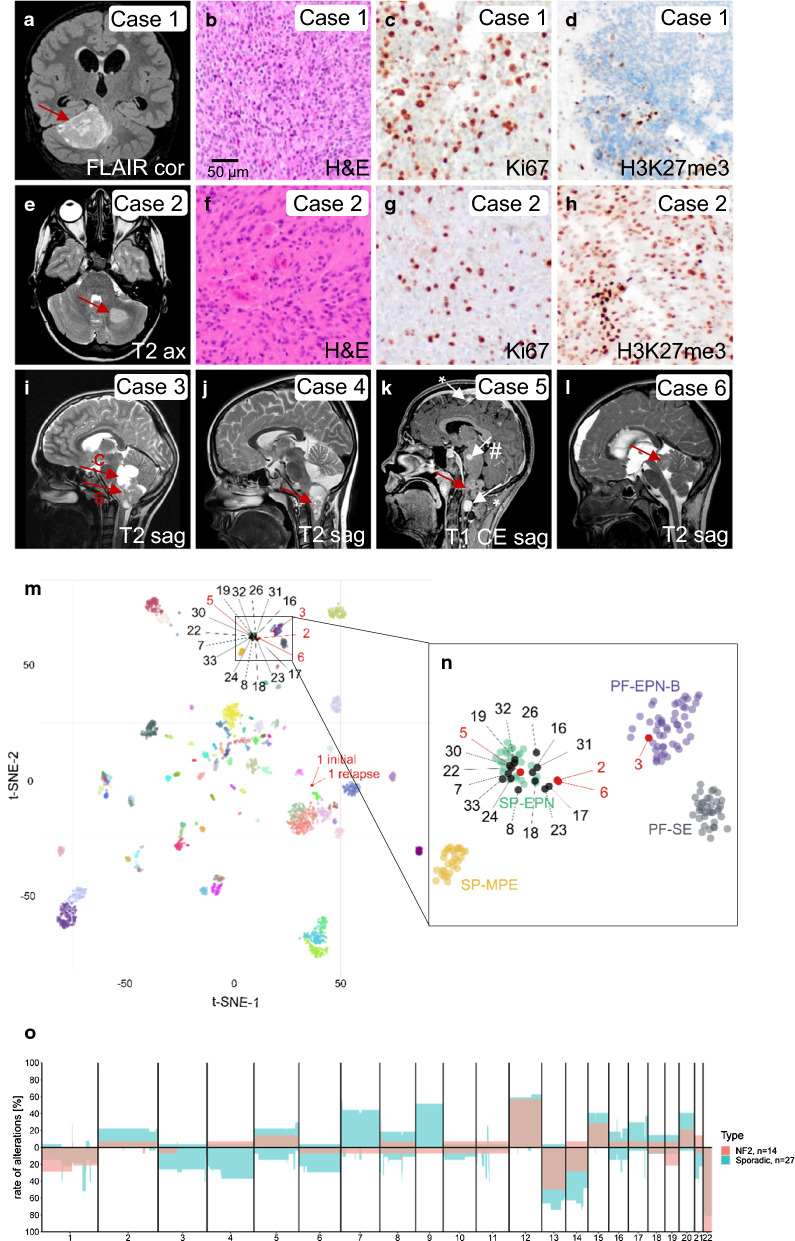
Ependymomas in NF2 patients. MRIs demonstrate intracranial localization of cases 1–6 (**a**, **e**, **i**–**l**). Red arrows indicate ependymomas, white arrows meningiomas (*) or schwannomas (#). Representative histology lacks typical pseudorosettes (**b**, **f**), but shows high proliferation in cases 1 and 2 (**c**, **g**). H3K27 trimethylation was lost in case 1 (**d**), but retained in case 2 (**h**). *T*-SNE plot (**m**) including all DNA methylation classes published by Capper et al. 2018 [1] shows that case 1 is unrelated to any of the reference classes. Sample 3 clearly falls into the class of PF-EPN-B (**n**). Intracranial cases 2, 5, and 6 as well as all 14 spinal tumors fell into the class of SP-EPN. Cumulative copy number variation profiles from reference SP-EPN (*n* = 27) [1] and NF2-associated SP-EPN (*n* = 14) suggest less chromosomal aberrations in NF2-associated cases (**o**). *ax* axial, *c* cystic, *cor* coronal, *s* solid, *sag* sagittal, *FLAIR* fluid-attenuated inversion recovery sequence, *CE* contrast enhancement

## Supplementary Information

Below is the link to the electronic supplementary material.

Supplementary file1 (PPTX 23752 KB)
