# Supplementary material for: Neurofibromatosis type 2 predisposes to ependymomas of various localization, histology, and molecular subtype
Source: Acta Neuropathol. 2021 Apr 12;141(6):971–4. doi: 10.1007/s00401-021-02304-4 (PMC8113165; doi:10.1007/s00401-021-02304-4)
Supplement: Supplementary file 1 — Supplementary file1 (PPTX 23752 KB) [file 401_2021_2304_MOESM1_ESM.pptx]

## Slide 1
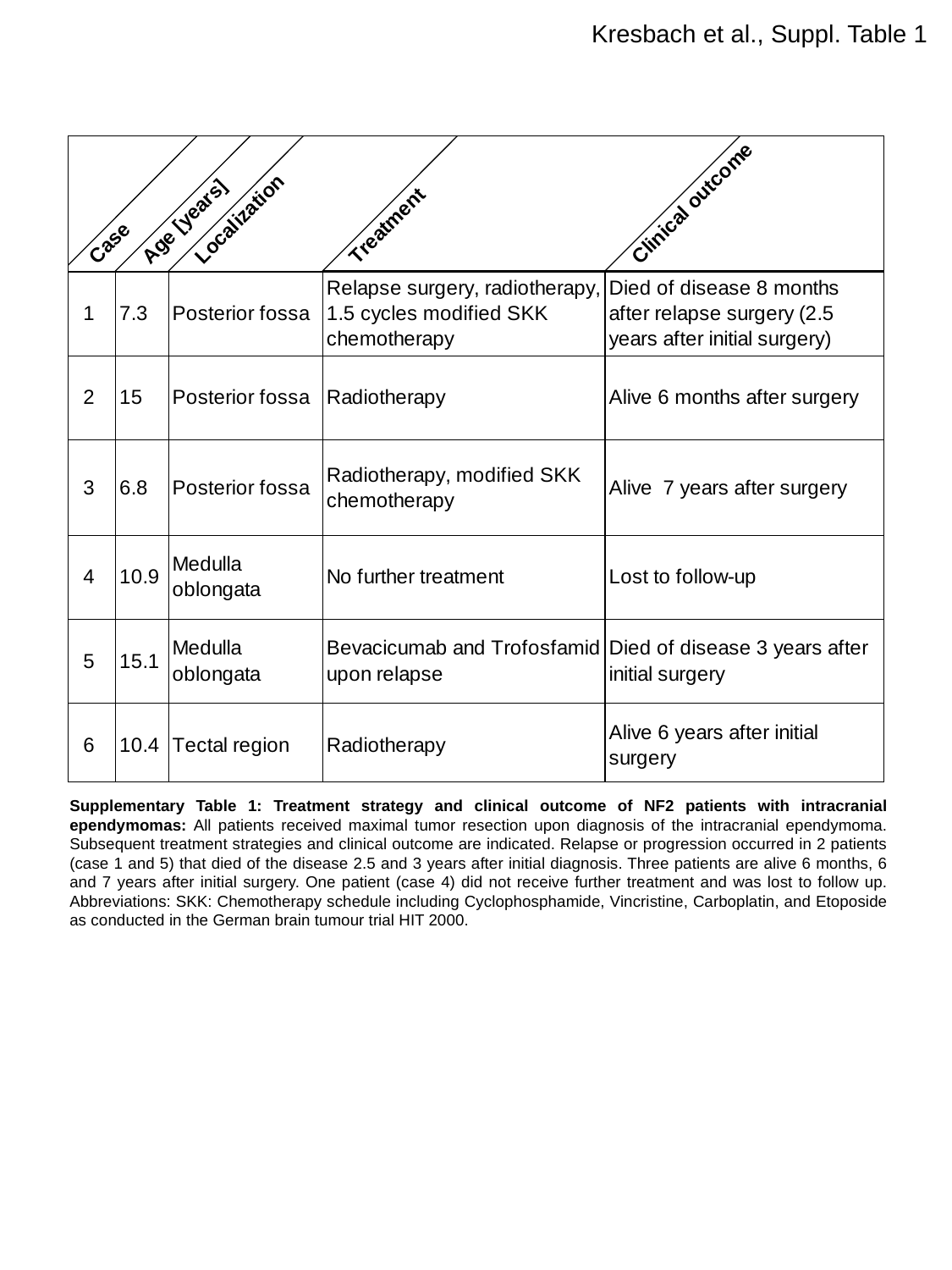

Kresbach et al., Suppl. Table 1
Supplementary Table 1: Treatment strategy and clinical outcome of NF2 patients with intracranial ependymomas: All patients received maximal tumor resection upon diagnosis of the intracranial ependymoma. Subsequent treatment strategies and clinical outcome are indicated. Relapse or progression occurred in 2 patients (case 1 and 5) that died of the disease 2.5 and 3 years after initial diagnosis. Three patients are alive 6 months, 6 and 7 years after initial surgery. One patient (case 4) did not receive further treatment and was lost to follow up. Abbreviations: SKK: Chemotherapy schedule including Cyclophosphamide, Vincristine, Carboplatin, and Etoposide as conducted in the German brain tumour trial HIT 2000.

## Slide 2
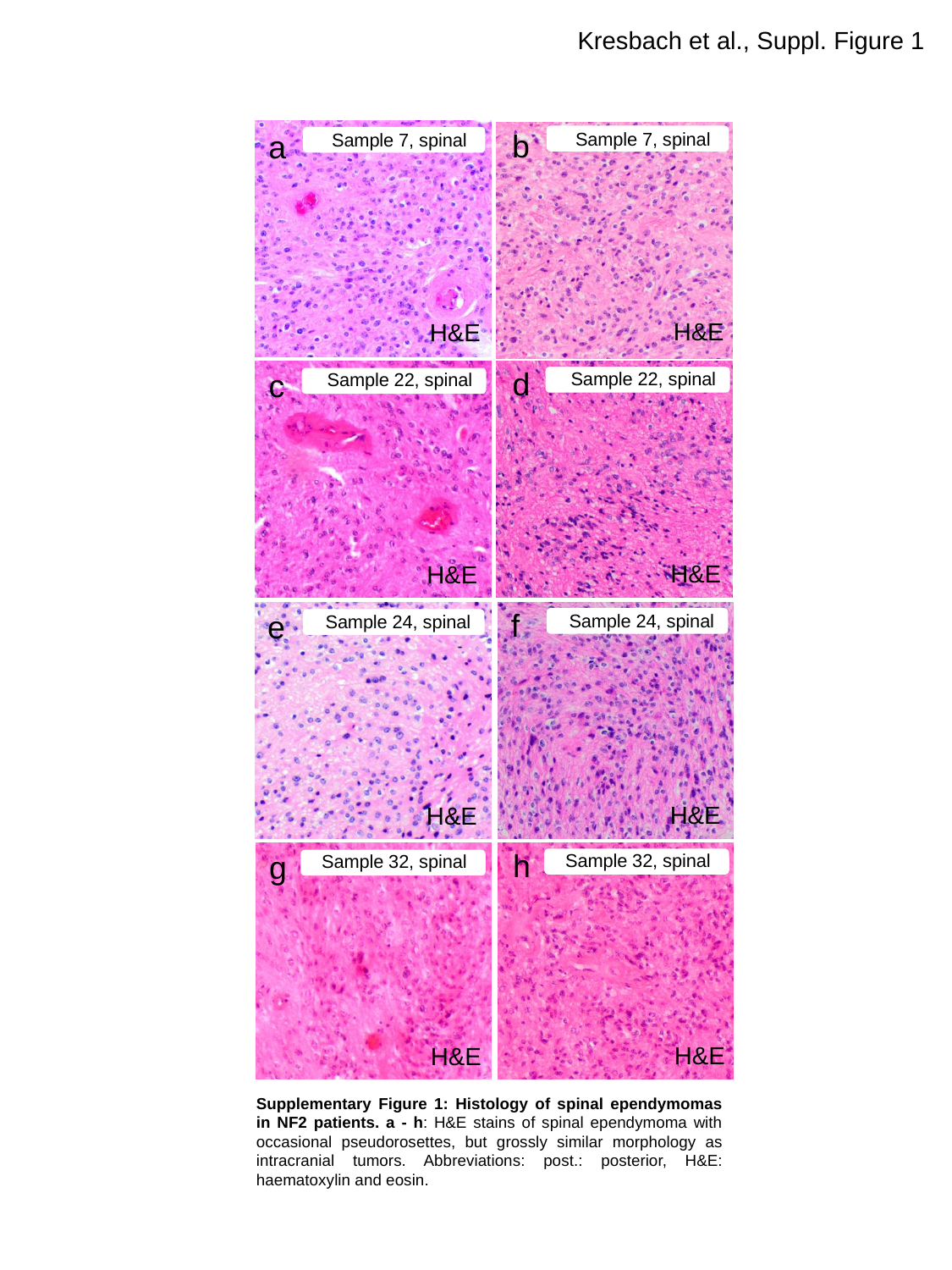

Kresbach et al., Suppl. Figure 1
b
Sample 7, spinal
a
Sample 7, spinal
H&E
H&E
d
c
Sample 22, spinal
Sample 22, spinal
H&E
H&E
f
e
Sample 24, spinal
Sample 24, spinal
H&E
H&E
h
g
Sample 32, spinal
Sample 32, spinal
H&E
H&E
Supplementary Figure 1: Histology of spinal ependymomas in NF2 patients. a - h: H&E stains of spinal ependymoma with occasional pseudorosettes, but grossly similar morphology as intracranial tumors. Abbreviations: post.: posterior, H&E: haematoxylin and eosin.

## Slide 3
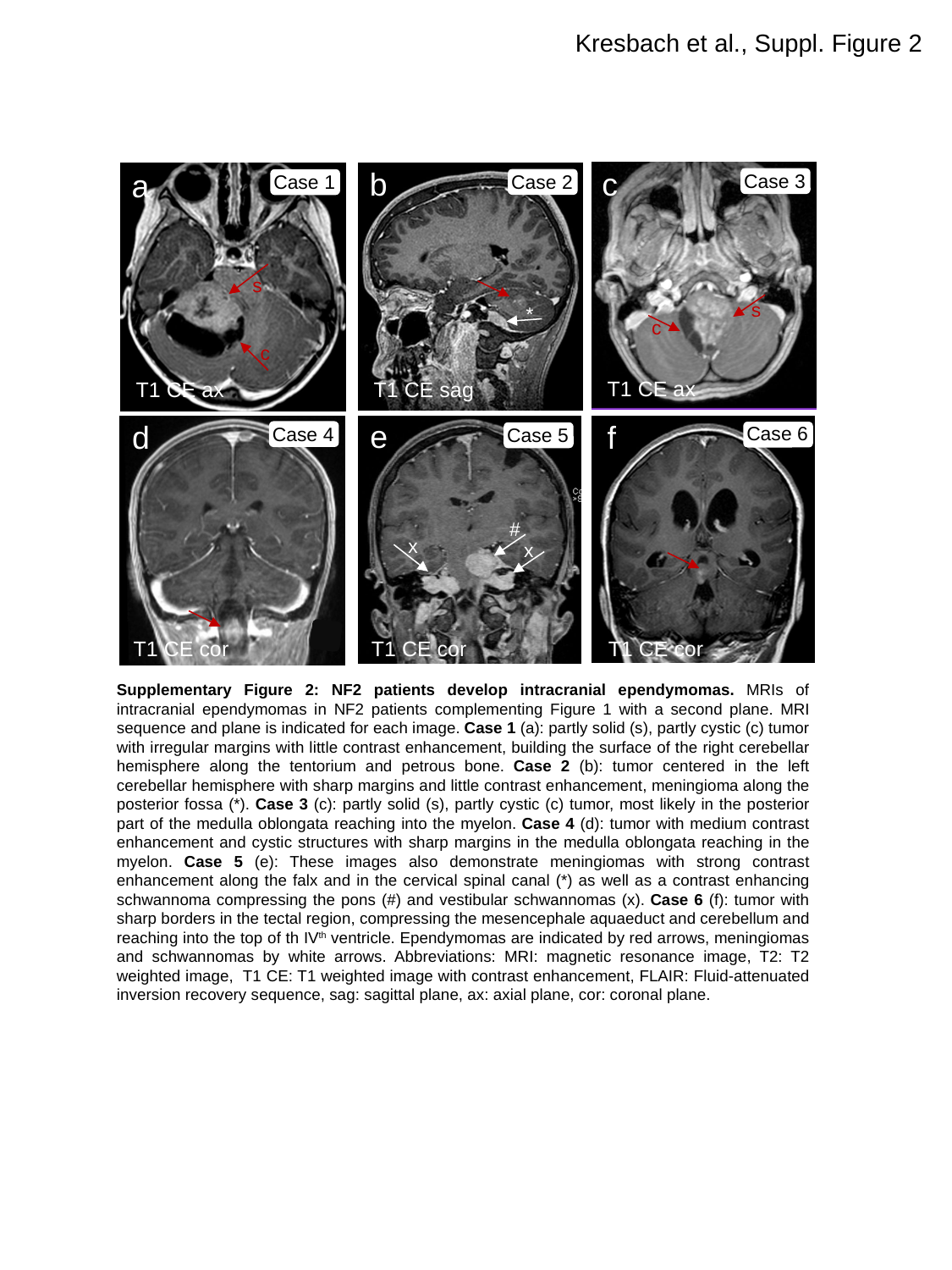

Kresbach et al., Suppl. Figure 2
b
c
Case 3
s
c
T1 CE ax
a
Case 1
s
c
T1 CE ax
Case 2
*
T1 CE sag
e
d
f
Case 6
Case 4
Case 5
#
x
x
T1 CE cor
T1 CE cor
T1 CE cor
Supplementary Figure 2: NF2 patients develop intracranial ependymomas. MRIs of intracranial ependymomas in NF2 patients complementing Figure 1 with a second plane. MRI sequence and plane is indicated for each image. Case 1 (a): partly solid (s), partly cystic (c) tumor with irregular margins with little contrast enhancement, building the surface of the right cerebellar hemisphere along the tentorium and petrous bone. Case 2 (b): tumor centered in the left cerebellar hemisphere with sharp margins and little contrast enhancement, meningioma along the posterior fossa (*). Case 3 (c): partly solid (s), partly cystic (c) tumor, most likely in the posterior part of the medulla oblongata reaching into the myelon. Case 4 (d): tumor with medium contrast enhancement and cystic structures with sharp margins in the medulla oblongata reaching in the myelon. Case 5 (e): These images also demonstrate meningiomas with strong contrast enhancement along the falx and in the cervical spinal canal (*) as well as a contrast enhancing schwannoma compressing the pons (#) and vestibular schwannomas (x). Case 6 (f): tumor with sharp borders in the tectal region, compressing the mesencephale aquaeduct and cerebellum and reaching into the top of th IVth ventricle. Ependymomas are indicated by red arrows, meningiomas and schwannomas by white arrows. Abbreviations: MRI: magnetic resonance image, T2: T2 weighted image, T1 CE: T1 weighted image with contrast enhancement, FLAIR: Fluid-attenuated inversion recovery sequence, sag: sagittal plane, ax: axial plane, cor: coronal plane.

## Slide 4
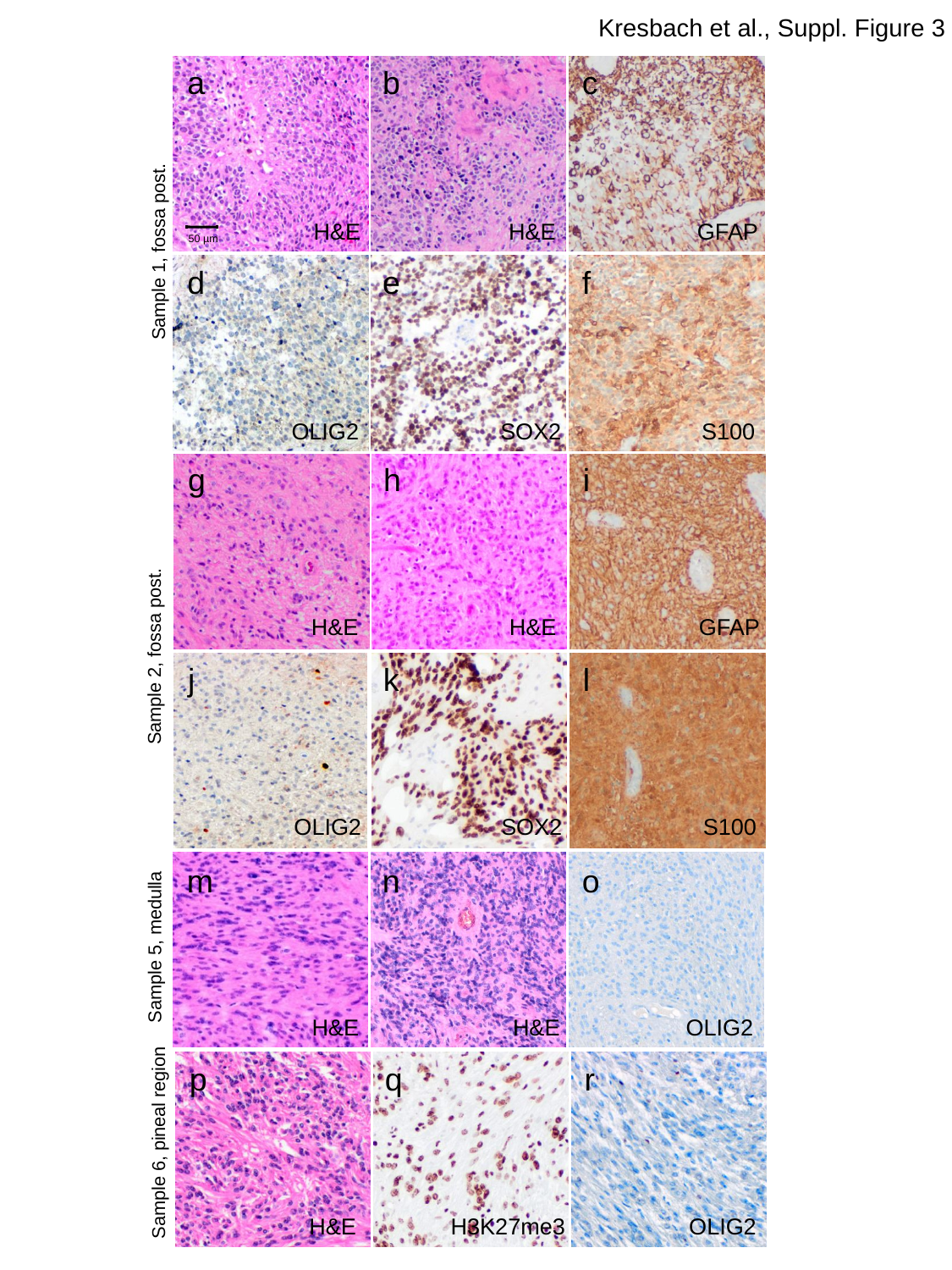

Kresbach et al., Suppl. Figure 3
a
b
c
H&E
H&E
GFAP
Sample 1, fossa post.
d
e
f
OLIG2
SOX2
S100
50 µm
g
h
i
H&E
H&E
GFAP
Sample 2, fossa post.
j
k
l
OLIG2
SOX2
S100
m
n
o
Sample 5, medulla
H&E
H&E
OLIG2
p
q
r
H&E
H3K27me3
OLIG2
Sample 6, pineal region

## Slide 5
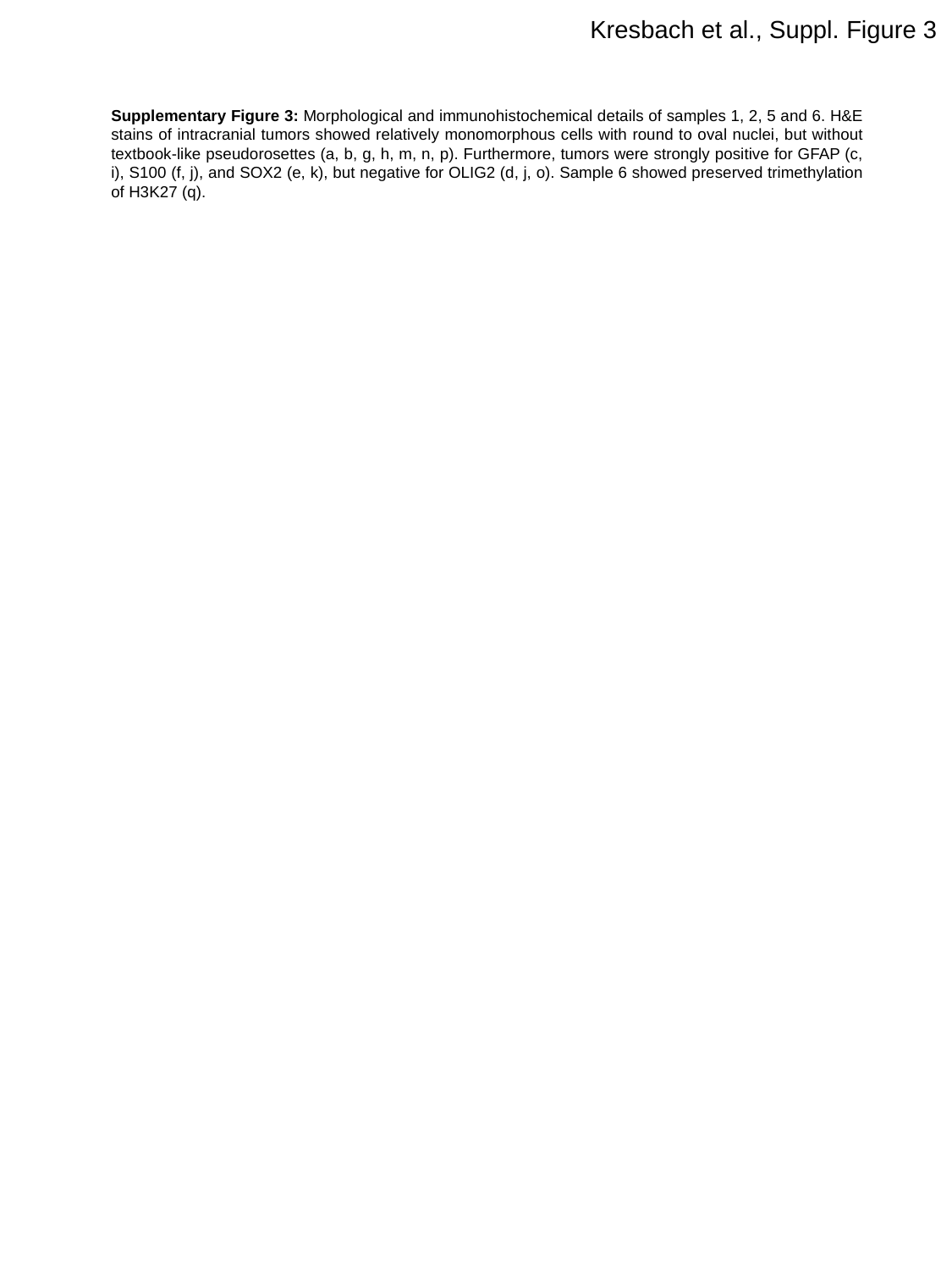

Kresbach et al., Suppl. Figure 3
Supplementary Figure 3: Morphological and immunohistochemical details of samples 1, 2, 5 and 6. H&E stains of intracranial tumors showed relatively monomorphous cells with round to oval nuclei, but without textbook-like pseudorosettes (a, b, g, h, m, n, p). Furthermore, tumors were strongly positive for GFAP (c, i), S100 (f, j), and SOX2 (e, k), but negative for OLIG2 (d, j, o). Sample 6 showed preserved trimethylation of H3K27 (q).
